# Ulinastatin Reduces the Resistance of Liver Cancer Cells to Epirubicin by Inhibiting Autophagy

**DOI:** 10.1371/journal.pone.0120694

**Published:** 2015-03-27

**Authors:** Bin Song, Qi Bian, Cheng Hao Shao, Gang Li, An An Liu, Wei Jing, Rui Liu, Yi-Jie Zhang, Ying-Qi Zhou, Xian-Gui Hu, Gang Jin

**Affiliations:** 1 Department of General Surgery, Changhai Hospital, Second Military Medical University, Shanghai, 200438, China; 2 Department of Nephrology, Changhai Hospital, Second Military Medical University, Shanghai, 200438, China; National Cheng Kung University, TAIWAN

## Abstract

During chemotherapy, drug resistance caused by autophagy remains a major challenge to successful treatment of cancer patients. The purpose of this study is to show that ulinastatin (UTI), a trypsin inhibitor, could reduce the resistance of liver cancer cells to chemotherapeutic agent epirubicin (EPI). We achieved this conclusion by analyzing the effect of EPI alone or UTI plus EPI on SMMC-7721 and MHCC-LM3 liver cancer cells. We also generated an EPI-resistant liver cancer cell line (MHCC-LM3er cells), and found that UTI could sensitize the LM3er cells to EPI. Autophagy usually functions to protect cancer cells during chemotherapy. Our study showed that UTI inhibited the autophagy induced by EPI in liver cancer cells, which promoted apoptosis, and therefore, reduced the resistance of the cancer cells to EPI. Further studies showed that the UTI-mediated inhibition on autophagy was achieved by inhibiting transcriptional factor nuclear factor-κB (NF-κB) signaling pathway. To verify our results in vivo, we injected MHCC-LM3 liver cancer cells or EPI-resistant LM3er cells into mice, and found that EPI could only effectively inhibit the growth of tumor in MHCC-LM3 cell-injected mice, but not in LM3er cell-injected mice. However, when UTI was also administered, the growth of tumor was inhibited in the MHCC-LM3er cell-injected mice as well. Our results suggest that UTI may be used in combination with anti-cancer drugs, such as EPI, to improve the outcome of cancer therapy.

## Introduction

Autophagy is an essential cellular process responsible for the degradation of damaged and dysfunctional cellular organelles and protein aggregates. This is a process conserved among yeast, plant, and animal cells and is important for providing sources of energy in response to nutrient stress and at critical times in development [1]. In general, autophagy is a tumor-suppression mechanism in normal cells and during early oncogenic transformation, but may also act as a critical survival pathway for established tumors. Developing and established tumors have high metabolic demands due to increased proliferation and high apoptotic thresholds. In this case, autophagy serves as a survival mechanism in many tumor cells, allowing them to escape apoptotic death in response to metabolic crisis. Many anti-cancer drugs are designed to chemically mimic nutrient deprivation and starvation, but autophagy is often up-regulated in response to the treatment by eliminating the damaged organelle to escape death, and therefore generates therapeutic resistance [2–4]. It has been shown that by increasing the production of reactive oxygen species in mitochondria, chemotherapeutic agents such as histone deacetylase inhibitors [5] and cisplatin [6] induce autophagy, and in many cases, the autophagic response to these treatments is cyto-protective [5]. Recently, it has been suggested that by combining with agents that disrupt autophagy, the effectiveness of anti-cancer drug may be enhanced [5].

Ulinastatin (UTI) is a glycoprotein that contains glycosaminoglycans and N-linked glycans. It inhibits the activities of a wide range of enzymes including trypsin, chymo-trypsin, kallikrein, plasmin, etc [7]. Clinically, UTI is mainly used to treat acute pancreatitis, chronic recurrent pancreatitis and acute circulatory failure [8, 9]. Recent studies have shown that UTI inhibits the growth of many types of cancers including gastrointestinal cancer and breast cancer, and may induce cell death if it is used together with other therapeutic agents [10–12]. In our preliminary studies, UTI was found to have inhibitory effect on autophagy. Since autophagy plays an important role in drug resistance in many types of cancer cells [13, 14], we therefore hypothesized that UTI might reduce drug resistance in cancer cells by inhibiting autophagy.

Epirubicin (EPI) is an anthracycline drug widely used in cancer chemotherapy [15]. It achieves the treatment by inducing apoptosis in cancer cells. However, at least in MCF-7 breast cancer cells, EPI also induces autophagy that protects the cancer cells by causing drug-resistance[15]. In this study, we investigated the effect of UTI on autophagy induced by EPI in liver cancer cells. We generated an EPI-resistant liver cancer cell lines (MHCC-LM3er cells) and studied the regulation of autophagy in these cells. We also studied the effect of UTI on autophagy and drug resistance in these cancer cells, and verified the results in vivo. It has been found that UTI suppressed autophagy induced by EPI, which was achieved by inhibiting the transcriptional factor nuclear factor-κB (NF-κB) signaling pathway. Our data suggest that UTI may be used as a therapeutic agent to assist cancer treatment by reducing drug resistance in cancer cells.

## Materials and Methods

### Antibody, Cell Lines, Plasmids and Other Materials

Mouse anti ACTB, rabbit anti p-IKKα, p-Nemo antibodies (Santa Cruz, USA) were used at 1:200 dilution. Rabbit anti LC3, mouse anti IKKα, mouse anti Nemo, and rabbit anti PARP, ATG5, ATG7,SQSTM1,BECN1 antibodies (Cell Signaling, USA) were used at 1:1,000 dilution. The HRP-labeled rabbit anti mouse EnVision labeling system was from Shanghai Changdao Biotech Inc.

Liver cancer cell lines SMMC-7721 and MHCC-LM3, and normal liver cells were gifts from Second Military Medical University, Shanghai. PcDNA3.1-GFP-LC3 was obtained from Eastern Hepatobiliary Surgery Hospital, Second Military Medical University, China. The cancer cell lines used in the study were purchased from Cell Bank of Type Culture Collection of Chinese Academy of Sciences, Shanghai Institute of Cell Biology, Chinese Academy of Sciences, where they were characterized by cell vitality detection, DNA-Fingerprinting, isozyme detection and mycoplasma detection. These cell lines were immediately expanded and frozen so that they could be restarted every 3 to 4 months from a frozen vial of the same batch of cells.

EPI (Eastern Hepatobiliary Surgery Hospital, Second Military Medical University, China) was used at the concentration of 2.5 μM. UTI (Techpool Bio-pharma, Guangdong, China) was used at the concentration of 1000 u/mL. Pyrrolidine dithiocarbamate (PDTC) (Beyotime Inc., Shanghai, China) was used at the concentration of 10 μM. Chloroquine (CQ, 10 μM) and 3-Methyladenine (3-MA, 10 mM) (Sigma-Aldrich, USA) were used for inhibition of autophagy. Mice were maintained and cared according to the university guidelines and Animal Use Protocols were approved by the Ethics Boards of the Eastern Hepatobiliary Surgery Hospital.

### Cell Culture, Transfection, Cell Growth Determination and Generation of Drug-Resistant MHCC-LM3 Cell Line

All liver cancer cells were cultured in DMEM with 10% FBS at 37°C in a 7% CO_2_ incubator, and transfected with Lipo2000 (Genechem Inc., Korea) at the confluence of 60–70%. Cell growth was determined with a MTS kit (Promega, USA) by following the manufacturer’s instruction. Drug-resistant MHCC-LM3 cell line was generated as described previously [15]. Briefly, MHCC-LM3 Cells were seeded in culture flasks and were allowed to reach ~ 80% confluence in fresh medium before being treated with EPI. The starting dose of EPI was 0.05 μM, and each following dose was 25–50% higher in concentration than the previous dose until the cells were stable in proliferation without significant cell death.

### Real-time PCR

Total RNA was extracted with RNeasy Mini kit and QIAshredder (Qiagen), and the first strand cDNA was synthesized with First-Strand cDNA Synthesis Kit (Abigen Biotechnology Co. Ltd.) by following the manufacturers’ instructions. Real-time PCR was performed with LightCycler 1.5 (Roche) by following the manufacturer’s protocol, and the following primers were used: for Beclin1: 5’-GGCTGAGGGATGGAAGGGTCTAAG-3’ and 5’-GTTTCGCCTGGGCTGTGGTAAGTA-3’; for Atg5: 5’-ATGACAGATGACAAAGATG-3’ and 5’-CTCATAACCTTCTGAAAGTG-3’; for At g7:5’-ATGGGGGACCCTGGACTGG-3’ and 5’-GCAGAGTCACCATTGTAG-3’; for P62: 5’-ACTGATGGCTGTAACGGTCTA-3’ and 5’-GGAAGCAGATGGCACAGAGG-3’; and for GAPDH: 5’-ACCACAGTCCATGCCATCAC-3’ and 5’-TCCACCACCCTGTTGCTGTA-3’.

### Apoptosis Assay

Cells washed with cold PBS were re-suspended in binding buffer (Beyotime Inc., Shanghai, China) and then supplemented with 2 μl Annexin-V-FITC (20 μg/ml) before being analyzed by flow cytometry.

### Autophagy Assays

Cells at 70–80% confluence were transfected with pcDNA3.1-GFP-LC3 with Lipo2000. The cells were then observed under fluorescence microscope and the cells with at least 3 green dots were considered autophagic cells. The percentage of autophagy = autophagic cells / total cells x 100%. For observation under electron microscope, cells washed with PBS were pelleted and the cell pellet was fixed in 2.5% glutaric dialdehyde and 1% osmic acid for 2 hr. After the wash, the pellet was dehydrated and embedded in epoxy resin. The ultra-thin sections (45 nm) were stained with lead acetate, and the stained sections were observed under JEM-2000EX electron microscope [16]. To monitor autophagic flux, the tandem mRFP-GFP-LC3 adenovirus construct obtained from Hanbio Inc (Shanghai, China) was used in this study. The exhibited green fluorescent protein (GFP) and red fluorescent protein (RRP) relied on different sensitivity of pH to monitor progression from the autophagic flux. In brief, mRFP-GFP-LC3 construct capitalized on the pH difference between the acidic autolysosome and the neutral autophagosome to monitor progression from the autophagosome to autolysosome by using confocal microscopy [17].

### Xenograft in Nude Mice

Male nude mice (4–6 week old and 15–20 g in weight, purchased from Institute of Material Medical of Shanghai, Chinese Academy of Medical Sciences) were maintained at 25~27°C with 45~50% humidity. Nude mice were maintained and cared according to the university guidelines and Animal Use Protocols were approved by the Ethics Boards of the Eastern Hepatobiliary Surgery Hospital. MHCC-LM3 cells and MHCC-LM3er drug-resistant cells were harvested and re-suspended in PBS at 5×10^6^ cells/0.2ml PBS. For each mouse, 200 μl of the cell suspension was injected under the skin on right shoulder. EPI (5mg/kg) and UTI (20000/mouse) were injected into tumor every 3 days.30 days later the mice were sacrificed for further research. The tumor sizes were calculated by using the formula V = ab^2^/2 (a: longest diameter of the tumor, b: shortest diameter).

## RESULTS

### UTI Inhibits Autophagy Induced By EPI

**Fig 1:**
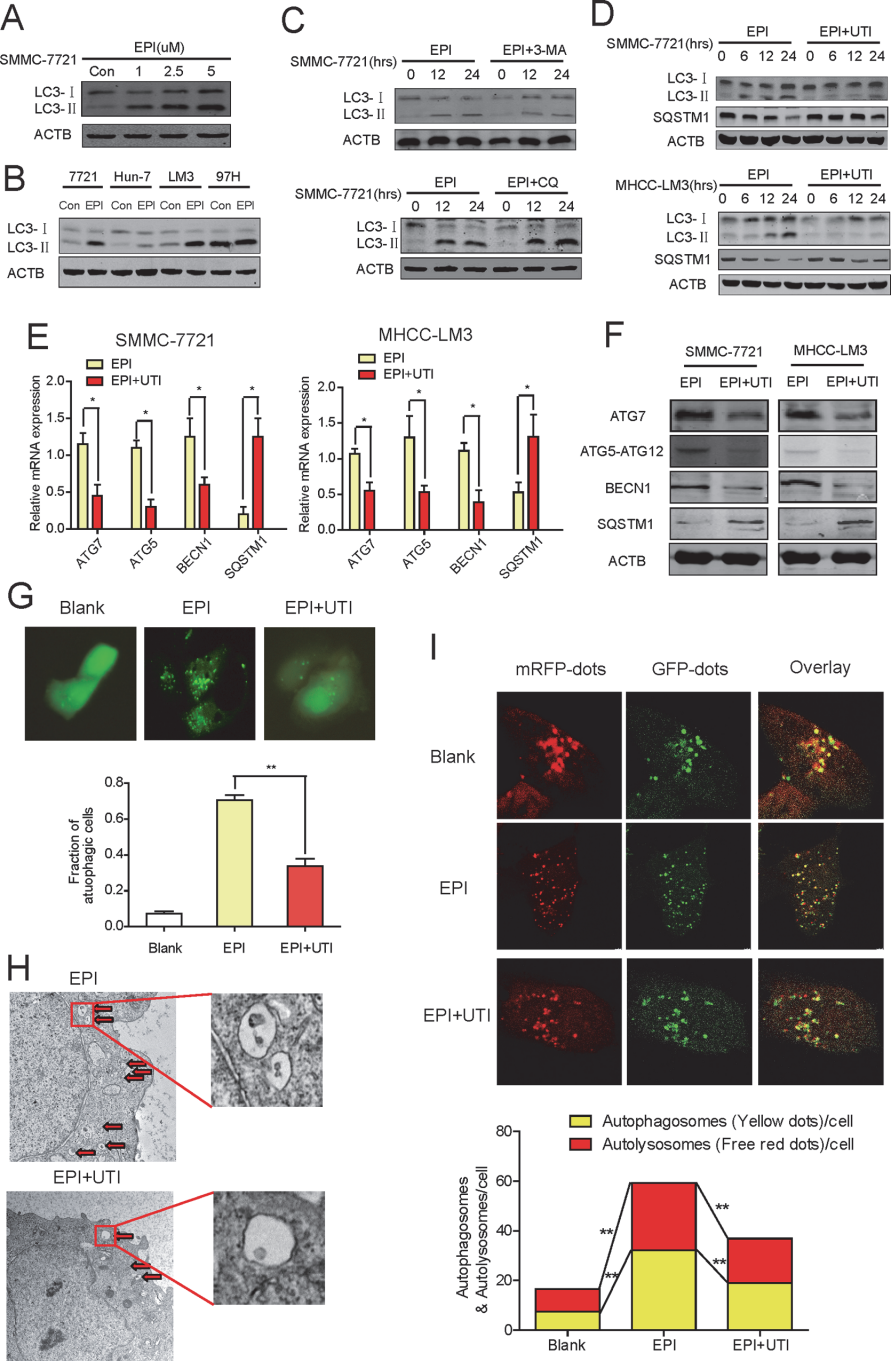
UTI inhibits autophagy induced by EPI in liver cancer cells. (A) The expression of LC3-II was detected in lysates of SMMC-7721 cells treated with different doses of EPI. (B) The LC3 level was detected in different liver cancer cells treated with EPI (2.5μM) or not for 24 hrs. (C) Western blot showed the level of LC3-II in SMMC-7721 cells treated with EPI (2.5μM) with/without CQ (10 μM)/3-MA (10 mM) at different time points. (D) Indicated molecules were detected with immunoblots in SMMC-7721 and MHCC-LM3 cells treated with EPI/EPI+UTI for indicated times. (E) Real-time PCR shows the mRNAs levels of autophagy-related genes in MHCC-LM3 and SMMC-7721 cells treated with EPI or EPI+UTI. (F) Western blot shows the protein levels of autophagy-related genes in MHCC-LM3 and SMMC-7721 cells treated with EPI/EPI+UTI for 24 hrs. (G) EPI/EPI+UTI were added to MHCC-LM3 cells for 24 hr after GFP-LC3 transfection. Upper panel, GFP-LC3 staining; lower panel, graph show the fraction of autophagic cells. (H) Electron micrograph of autophagic vesicles in MHCC-LM3 cells treated with EPI+EPI+UTI. The enlarged high resolution images of the square areas were shown. (I) MHCC-LM3 cells infected with GFP-mRFP-LC3 adenovirus for 24h, then treated withEPI/EPI+UTI. Representative images and graphs were shown. The experiment was repeated 3 times. (*P<0.05,**P<0.01).

EPI has been reported to induce autophagy in breast cancer cells and causes drug resistance[15]. To verity if it has the similar effect in liver cancer cells, we treated SMMC-7721 cells with EPI at various concentrations to determine the range that EPI could effectively induce autophagy [15, 18]. As shown in Fig 1A, autophagy increased in an EPI concentration-dependent manner. Based on this result, we chose a moderate concentration of 2.5 μM for subsequent studies. Next, we checked that whether EPI induced autophagy in other liver cancer cells. EPI could significantly enhance autophagy in SMMC-7721, Huh-7 and HCC-LM3 cells and mildly in 97H cells (Fig 1B). It has been shown that CQ could inhibit the infusion of autophagosomes and lysosomes, resulting in the accumulation of autophagosomes and LC3-II expression. Meanwhile, 3-MA inhibits class I as well as class III PtdIns3Ks, which is necessary for autophagosomes formation [16]. With the two autophagy inhibitors, we validated that EPI could enhance the whole autophagy process, including autophagosomes and autolysosomes formation (Fig 1C). Next, we wanted to know if the EPI-induced autophagy could be inhibited by UTI. For this purpose, we treated SMMC-7721 and MHCC-LM3 cells with EPI or EPI+UTI, the increase of LC3-II and decrease of SQSTM1 were significantly inhibited by EPI+UTI treatment, suggesting that the EPI-induced autophagy was inhibited by the addition of UTI (Fig 1D). This result was further supported by the observation that the ATG7, ATG5 and BECN1 expressions decreased and SQSTM1 expression increased both at the mRNA level (Fig 1E) and protein level (Fig 1F) in cells being treated with EPI+UTI comparing with the cells being treated with EPI alone. In the cells transfected with pcDNA3.1-GFP-LC3, the expression of GFP-LC3 (green dots) were also reduced significantly in the cells being treated with EPI+UTI comparing with that in the cells being treated with EPI alone (Fig 1G). Furthermore, comparing with the cells being treated with EPI, the amount of autophagic vesicles in cells being treated with EPI+UTI was also reduced significantly as observed under electron microscope (Fig 1H). To further confirm the function of UTI on autophagic flux, we check the number of autophagosomes and autolysosomes in MHCC-LM3 cells treated with either EPI or EPI+UTI, UTI could significantly reduce the autophgic flux (Fig 1I). Together, these results strongly suggest that UTI inhibits EPI-induced autophagy in liver cancer cells.

### UTI Promotes Apoptosis Induced by EPI

**Fig 2:**
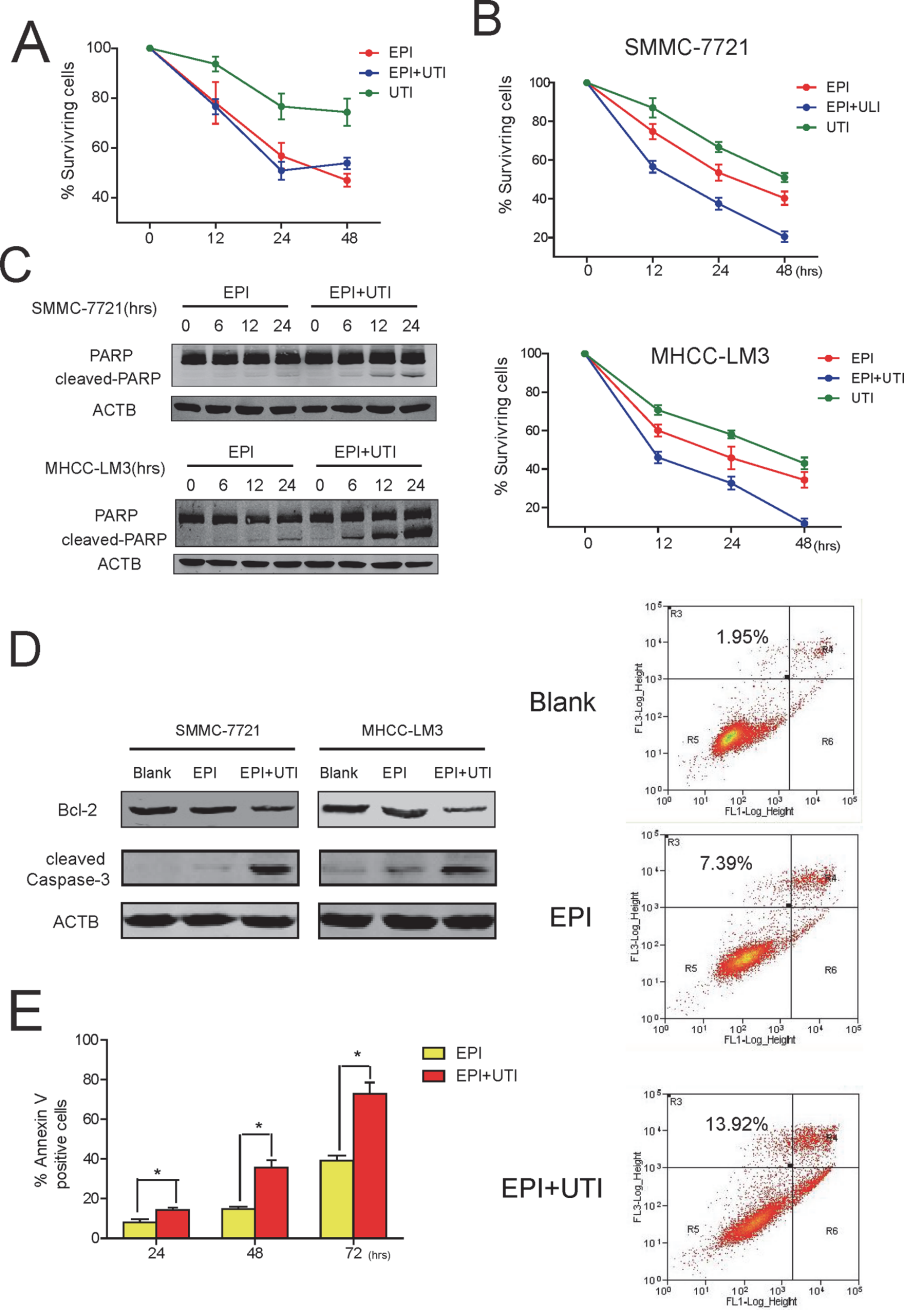
Ulinastatin promotes epirubicin-induced apoptosis in liver cancer cells. (A) Percentage of surviving L02 cells treated with EPI/EPI+UTI/UTI. (B) Percentage of surviving SMMC-7721 and MHCC-LM3 liver cancer cells under the treatment of EPI/EPI+UTI/UTI for indicated times.(C, D) Western blot of indicated molecules were shown in SMMC-7721 and MHCC-LM3 liver cancer cells under the treatment of EPI/EPI+UTI (Treatment in D for 24 hr). ACTB was blotted as a loading control. (E) ANNEXIN V staining shows the percentage of cells underwent apoptosis under the treatment of EPI/EPI+UTI for indicated times. Left panel was average result of 3 experiments; right panel was representative result. Results are average of 3 independent experiments, (*P<0.05,**P<0.01).

As a chemotherapy drug, EPI eliminates cancer cells by inducing apoptosis [19]. Since we have shown that UTI inhibits autophagy induced by EPI, we suspect that UTI might also promote cancer apoptosis in these cells. To find out if UTI has any effect on apoptosis induced by EPI, we first checked whether the combination of EPI and UTI could cause toxic effect in normal liver cell. As shown in Fig 2A, there was no significant difference in Surviving rates between EPI group and EPI+UTI group, suggesting that EPI+UTI would not cause more cell death on normal liver cells. Next, we examined the amount of the SMMC-7721 and MHCC-LM3 liver cancer cells treated with EPI or EPI+UTI, and found that the cell numbers were significantly decreased if both EPI and UTI were added (Fig 2B). The cleavage of PARP, an indicator of apoptosis, was also enhanced in cells treated with EPI+UTI comparing with that in the cells treated with EPI alone (Fig 2C). Furthermore, the treatment with EPI+UTI resulted in decreased level of apoptosis inhibitor Bcl-2 and increased cleavage of caspase-3 (Fig 2D). In supportive of this result, Annexin V staining showed that the percentage of cells underwent apoptosis was higher in cells treated with EPI+UTI (Fig 2E). Taken together, these results strongly suggest that UTI promotes apoptosis when it is used in combination with EPI, and could potentially enhance the effectiveness of EPI in cancer treatment.

### UTI Promotes EPI-Induced Apoptosis in Liver Cancer Cell by Inhibiting Autophagy

**Fig 3:**
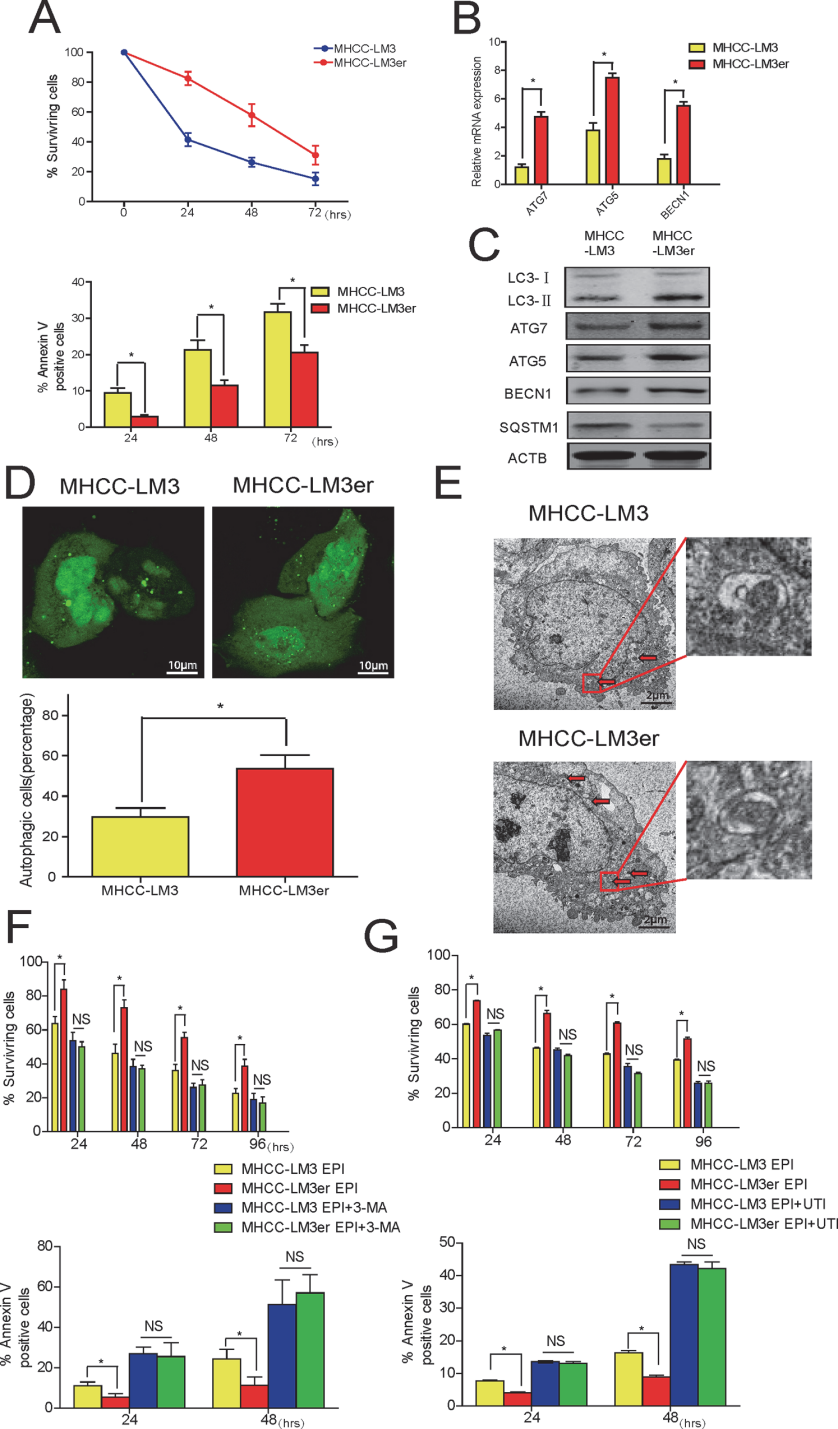
Ulinastatin promotes epirubicin-induced apoptosis in liver cancer cells by inhibiting autophagy. (A) Percentage of surviving MHCC-LM3 and LM3er cells under the treatment of EPI. Lower panel showed the percentages of Annexin V-positive MHCC-LM3 or MHCC-LM3er cells. (B) Real-time PCR showed the mRNA levels of ATG5, ATG7 and BECN1 in MHCC-LM3 and MHCC-LM3er cells. (C) Western blot of LC3-I, LC3-II, ATG7, ATG5, BECN1 and SQSTM1 in LM3 and LM3er cells. ACTB was a loading control. (D) GFP-LC3 dots in MHCC-LM3 and LM3er cells. Upper panel, immunofluorescence microscopy showed the representative image of GFP-LC3 dots, and lower panel was fraction of autophagic cells in MHCC-LM3 or MHCC-LM3er cells. (E) Electron micrograph of autophagic vesicles in MHCC-LM3 and LM3er cells. The enlarged high resolution images of the square areas were shown. (F) Percentage of surviving (upper panel) and ANNEXIN V-positive cells (lower panel) in MHCC-LM3 and LM3er cells under the treatment of EPI/EPI+3-MA or EPI/EPI+UTI. Results are average of 3 independent experiments (*P<0.05,**P<0.01).

Autophagy plays indispensable protective roles in EPI-induced cell death in cancer cells [15, 20]. Knowing that UTI promotes apoptosis induced by EPI, we wanted to know if this was achieved by inhibiting autophagy that was also induced by EPI. For this purpose, we generated an EPI-resistant cell line LM3er by applying low dose EPI to cell culture media over a period of time (see Materials and Methods). To both the MHCC-LM3er cells and the non EPI-resistant (MHCC-LM3) control cells, we added EPI and found that the number of surviving MHCC-LM3er cells was greater than that of the MHCC-LM3 cells, and fewer MHCC-LM3er cells underwent apoptosis than the control cells (Fig 3A). This result indicated that the EPI-resistant cells (MHCC-LM3er) were better protected from EPI-induced apoptosis. To find out if the resistance to EPI was due to increased autophagic activity in MHCC-LM3er cells, we examined the basal expression of several autophagy markers and found that in MHCC-LM3er cells, the mRNA levels of ATG7, ATG5 and BECN1 were higher than that in control cells (Fig 3B). We also examined the basal levels of several autophagy-related proteins, and found that the levels of LC3-II, ATG7, ATG5 and BECN1 increased in MHCC-LM3er cells as well, whereas the level of SQSTM1 decreased (Fig 3C). Fluorescence microscopy also showed that comparing with the control cells, there were more autophagic cells in MHCC-LM3er group (Fig 3D). Furthermore, under electron microscope, we found more autophagic vesicles in the MHCC-LM3er cells (Fig 3E). Thus, these results suggest that the EPI-resistant MHCC-LM3er cells have higher basal autophagic activity than that in the control cells. Furthermore, we found that MHCC-LM3er cells could resist to EPI-induced cell death comparing with MHCC-LM3 cells, and inhibition of autophagy with 3-MA could completely block the resistance of MHCC-LM3er cells to EPI. The similar results were also obtained when UTI was used (Fig 3F and 3G). This result strongly suggests that UTI promotes EPI-induced apoptosis in liver cancer cells by inhibiting autophagy.

### UTI Regulates Autophagy by Inhibiting NF-κB Signaling Pathway

**Fig 4:**
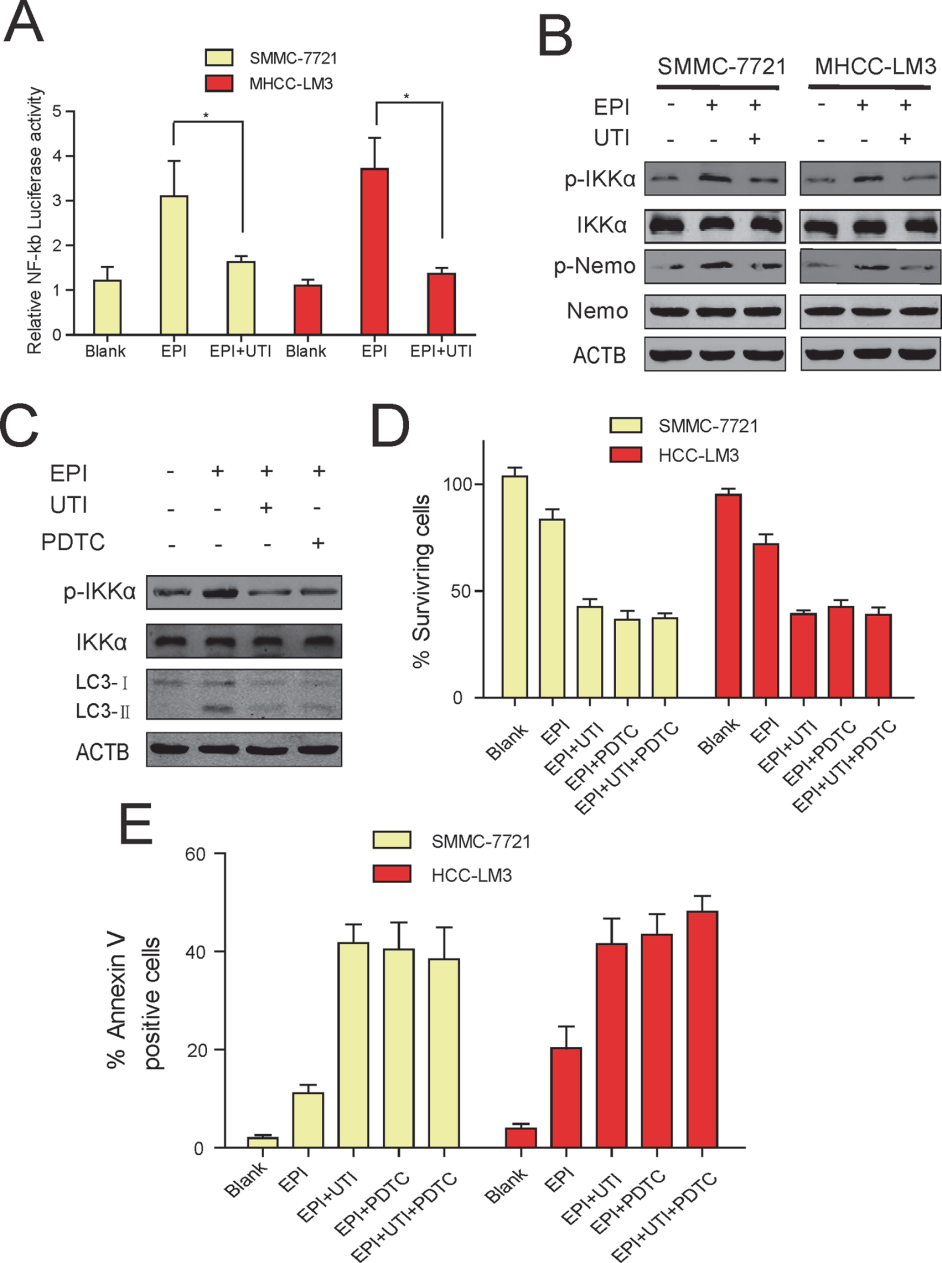
Ulinastatin regulates autophagy by inhibiting NF-κB signal pathway. (A) The relative NF-κB activity in SMMC-7721 and MHCC-LM3 cells under the treatment of EPI/EPI+UTI for 24 hr. (B) Western blots of p-IKK alpha and p-Nemo in comparison with total protein levels of IKK alpha and Nemo, respectively, in SMMC-7721 and MHCC-LM3 liver cancer cells under the treatment of EPI/EPI+UTI for 24 hr. ACTB is a loading control. (C) Western blot of p-IKK alpha, total IKK alpha, LC3-I and LC3-II in MHCC-LM3 cells under the treatment of EPI/EPI+UTI/EPI+PDTC for 24 hr (D, E) Percentage of surviving (D) and ANNEXIN V-positive cells (E) in SMMC-7721 and MHCC-LM3 cells under the treatment of EPI/EPI+UTI/EPI+PDTC/EPI+UTI+PDTC for 24 hr. Results are the average of 3 independent experiments (*P<0.05, **P<0.01).

NF-κB signaling pathway positively regulates autophagy [14, 21, 22]. UTI could inhibit the activation NF-κB signaling pathway [23, 24]. We therefore speculated that UTI might inhibit autophagy in the liver cancer cells by suppressing the NF-κB signaling pathway. To test this hypothesis, we treated SMMC-7721 and MHCC-LM3 cells with EPI alone or EPI+UTI, and found that the activity of NF-κB was suppressed in UTI supplemented cells (Fig 4A). This result was supported by the inhibition on the phosphorylation of IKK-α and Nemo (Fig 4B). Moreover, we found that the addition of PDTC, an inhibitor of NF-κB signaling pathway, to MHCC-LM3 cells inhibited the phosphorylation of IKK-α and the expression of LC3-II (Fig 4C). These results suggest that UTI inhibits autophagy by suppressing the NF-κB signaling pathway. To find out if the addition of the drugs, UTI or PDTC, promoted the epirubicin-induced apoptosis in the liver cancer cells, we added EPI, EPI+UTI, EPI+PDTC, or EPI+UTI+PDTC to SMMC-7721 and MHCC-LM3 liver cancer cells, and observed similar percentage of surviving cells in EPI+UTI, EPI+PDTC or EPI+UTI+PDTC supplemented cells. Particularly, there was no added effect on cell death if both UTI and PDTC are added, suggesting that both UTI and PDTC promote apoptosis in a same mechanism, which is to inhibit the NF-κB signaling pathway-regulated autophagy (Fig 4D and 4E).

### UTI Also Promotes Apoptosis by Inhibiting NF-κB Signaling Pathway-Induced Autophagy in Vivo

**Fig 5:**
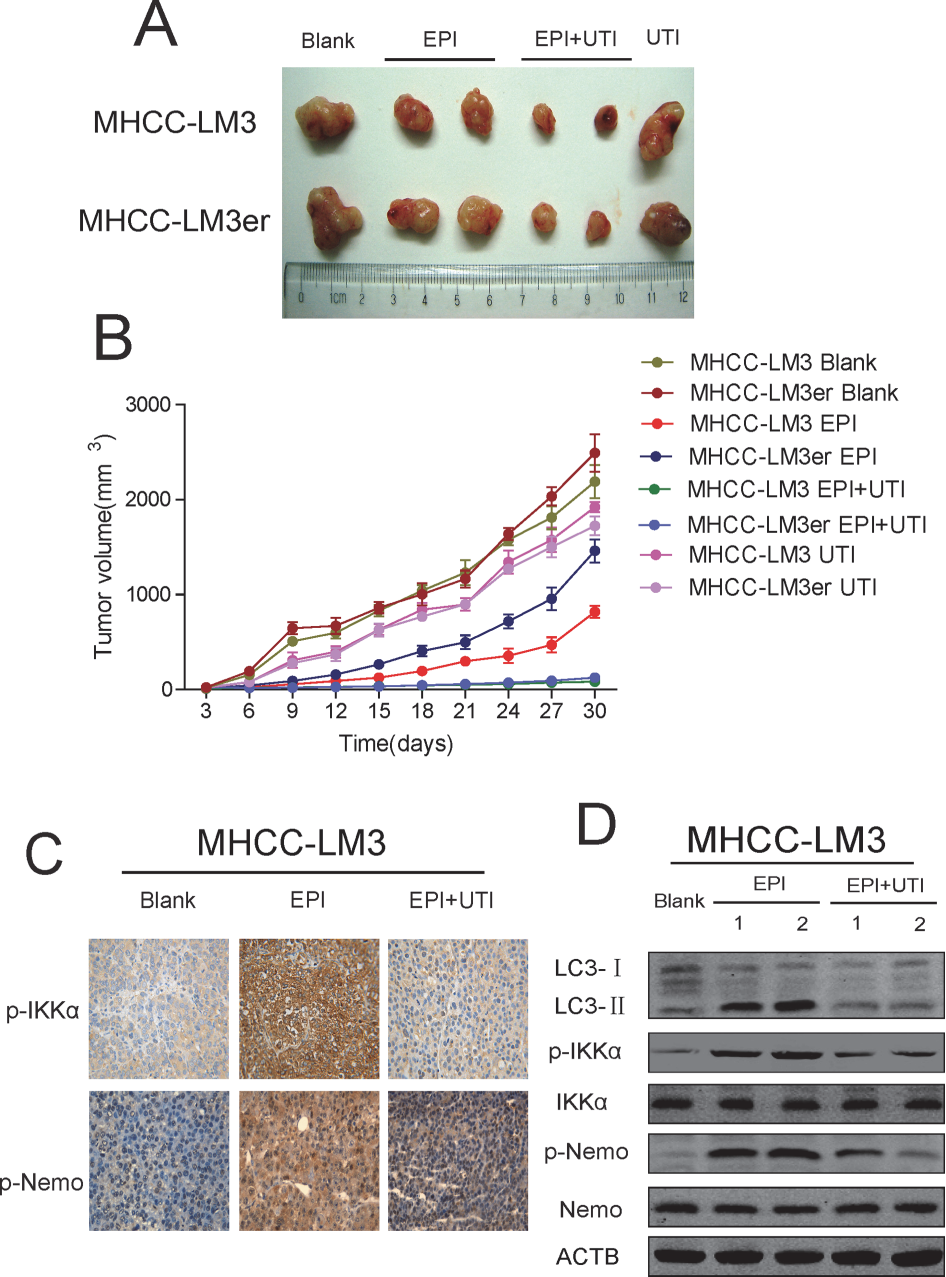
In vivo study verifies that ulinastatin regulates autophagy by inhibiting NF-κB signaling pathway. (A, B) Tumor sizes (A) and tumor growth curve (B) from mice xenografted with MHCC-LM3 or MHCC-LM3er cells (n = 6) treated with EPI/EPI+UTI. The growth curve was obtained from multiple measurements of different mice in the same group. (C) Histoimmunochemical analysis shows the expression of p-IKKα and p-Nemo in xenograft mice under the treatment of EPI/EPI+UTI. (D) Western blot of p-IKKα and p-Nemo in samples from xenograft mice under the treatment of EPI/EPI+UTI. ACTB is a loading control. Results are the average of 3 independent experiments (*P<0.05, **P<0.01).

To prove our above result in vivo, we injected MHCC-LM3 or EPI-resistant MHCC-LM3er cells under the skin in mice (n = 6), and found that administration of EPI could inhibit the growth of tumor in MHCC-LM3 cell-injected mice, but to a less extent in MHCC-LM3er cell-injected mice. However, when UTI was administered, the tumor growth in the MHCC-LM3er cell-injected mice was inhibited, and the growth rate was similar to that in the MHCC-LM3 cell-injected mice (Fig 5A and 5B). To find out if UTI inhibited the NF-κB signaling pathway in vivo, we administered EPI alone or EPI+UTI to the mice and analyzed the activity of NF-κB signaling pathway in the tumors. It was found that comparing to control mice, the level of LC3-II and the phosphorylations of IKKα and Nemo were all reduced in the tumors of mice administered with both EPI+UTI, indicating the inhibition of NF-κB signaling pathway (Fig 5C and 5D). This result suggests that UTI promotes apoptosis by inhibiting NF-κB signaling pathway-regulated autophagy in vivo.

## DISCUSSION

Regardless of cancer type, drug resistance remains a major challenge to successful chemotherapeutic treatment for cancer patients [25]. It is clearly important to identify and develop novel therapeutic strategies to improve the outcome of cancer treatment. Autophagy is a double-edged sword in cancer, it functions as both anti- and pro-cancer mechanisms [26]. In liver cancer, autophagy can protect liver by suppressing inflammation, tissue damage and genome instability to inhibit cancer initiation; on the other hand, it is required for cancer cell survival and cancer development [26]. Many current cancer therapies, including chemotherapy and radiation-therapy, impose metabolic stress on cancer cells and induce autophagy, which in turn, protect the cells from apoptosis and cause drug resistance. Therefore, the use of autophagic inhibitors in combination with therapeutic agents may particularly enhance the therapeutic efficacy [25, 27].

UTI is an important inhibitor to trypsin, and recent studies have shown that it works with other chemotherapeutic agents to promote apoptosis, resulting in the inhibition of tumor growth at least partially due to its inhibitory effect on many signaling pathways involved in the growth [10, 11, 28–30]. In our preliminary studies, we found that UTI was inhibitor to autophagy, and therefore, hypothesized that it might also improve cancer therapy by inhibiting autophagy and reduce resistance to chemotherapeutic drugs in cancer cells. Indeed, our data showed that in liver cancer cells, UTI inhibited the elevation of autophagy induced by EPI, a widely used cancer chemotherapeutic drug. When it was used in combination with EPI, UTI promoted apoptosis in the cells. This result suggested that in liver cancer cells, UTI alone had minimal effect on cell proliferation, but when it was applied together with EPI, it enhanced the EPI-induced apoptosis, most likely by inhibiting autophagy (Fig 2B). This result was further supported by the observation that in EPI-resistant cells, UTI was able to effectively sensitize the cells to EPI by inhibiting autophagy (Fig 3).

Since NF-κB is reported to positively regulate autophagy, we tested if UTI inhibited autophagy by suppressing NF-κB signaling pathway. Our results showed that the addition of both EPI and UTI to liver cancer cells significantly inhibited the NF-κB luciferase activity, and reduced the phosphorylation of IKK-α and Nemo, a result has also been verified by in vivo studies. These results strongly suggest that the NF-κB signaling pathway is inhibited when both EPI and UTI are applied. To prove that the suppression of NF-κB signaling pathway would lead to the inhibition of autophagy, we inhibited the NF-κB signaling pathway by adding PDTC, a known inhibitor of NF-κB signaling pathway, to the cells together with EPI, and observed the inhibition of autophagy as expected. This result strongly supports our hypothesis that like PDTC, UTI could inhibit autophagy by inhibiting the NF-κB signaling pathway. To further prove that both UTI and PDTC acted on the same signaling pathway, rather than worked independently to regulate apoptosis, we added both PDTC and UTI together with EPI to cancer cells, but did not observe any further increase of apoptosis activity. This result strongly supports our conclusion that UTI and PDTC did not work synergistically, and UTI alone was as effective as PDTC in inhibiting the NF-κB signaling pathway to promote apoptosis.

Autophagy-induced drug resistance in cancer cells has been a challenge to cancer treatment [31]. Anti-autophagy strategies have been used in many clinical trials registered with the National Cancer Institute in USA in a variety of human cancers [32]. However, CQ or HCQ (Hydroxychloroquine) were used to inhibit the infusion of the autophagosomes and lysosomes in most of clinical trials, the use of other autophagy inhibitors that could restrain the formation of autophagosomes in the clinical trials have not been reported. UTI has been used for the treatment of other diseases such as acute pancreatitis, and our results have demonstrated that UTI could inhibit EPI-induced autophagy and promote apoptosis during cancer therapy, most likely by inhibiting the NF-κB signaling pathway. EPI is a widely used cancer chemotherapy agent, but it can also cause drug-resistance by inducing autophagy. Our finding implies that UTI may be used in combination with anti-cancer drugs such as EPI to improve the outcome of cancer therapy. In addition, the results may also shed lights on the application of UTI in other clinical fields.
